# Conceptual and quantitative categorization of wave-induced flooding impacts for pedestrians and assets in urban beaches

**DOI:** 10.1038/s41598-023-32175-6

**Published:** 2023-05-04

**Authors:** J. L. Garzon, Ó. Ferreira, M. T. Reis, A. Ferreira, C. J. E. M. Fortes, A. C. Zózimo

**Affiliations:** 1 CIMA, Centre of Marine and Environmental Research\ARNET - Infrastructure Network in Aquatic Research, University of Algarve, Campus de Gambelas, 8000-139, Faro, Portugal; 2 Hydraulics and Environment Department, National Laboratory for Civil Engineering, Av. Do Brasil, 101, 1700-066 Lisboa, Portugal

**Keywords:** Natural hazards, Civil engineering

## Abstract

Beaches combined with sloping structures are frequently the first element of defense to protect urban areas from the impact of extreme coastal flooding events. However, these structures are rarely designed for null wave overtopping discharges, accepting that waves can pass above the crest and threat exposed elements in hinterland areas, such as pedestrians, urban elements and buildings, and vehicles. To reduce risks, Early Warning Systems (EWSs) can be used to anticipate and minimize the impacts of flooding episodes on those elements. A key aspect of these systems is the definition of non-admissible discharge levels that trigger significant impacts. However, large discrepancies in defining these discharge levels and the associated impacts are found among the existing methods to assess floodings. Due to the lack of standardization, a new conceptual and quantitative four-level (from no-impact to high-impact) categorization of flood warnings (EW-Coast) is proposed. EW-Coast integrates and unifies previous methods and builds on them by incorporating field-based information. Thus, the new categorization successfully predicted the impact level on 70%, 82%, and 85% of the overtopping episodes affecting pedestrians, urban elements and buildings, and vehicles, respectively. This demonstrates its suitability to support EWSs in areas vulnerable to wave-induced flooding.

## Introduction

Coastal flooding is a major threat to communities living in low-lying areas and the increase in the anthropogenic pressure in coastal zones and the effects of climate change (e.g., sea-level rise, increase in storminess and its frequency) are promoting an enhancement of the existing risks for population and properties^1–4^. Coastal flooding results from the interaction of oceanic and atmospheric processes with the local and regional features (topography, nearshore bathymetry, continental shelf, and land use). Among the different oceanic agents that might drive coastal flooding, wave-related processes have been found to be the dominant component in large areas of the globe compared to storm surges and tides^5^. When waves approach the shoreline, a large part of the wave energy is dissipated across the surf zone by wave breaking. However, a portion of the remaining energy is converted to potential energy in the form of wave runup on the beach foreshore^6^ contributing to boosting the extreme water levels^3^. When the existing natural or man-made coastal protection structure (constructed on land) is lower than the maximum level that water can reach by wave attack, a discharge occurs over the structure and propagates inland. It can be called green water^7^ (non-impulsive), when a layer of water passes over the crest, or white water^7^ (or impulsive conditions) when waves break on the seaward face of the structure and produce significant volumes of splash or spray (not considered here). Therefore, wave runup (and overtopping) is important to coastal planners and engineers because it delivers much of the energy responsible for causing a flooding event^8^. Besides disruptions in local services and transportation, during such events, seawater can travel with high velocities, which in turn can affect the integrity of urban elements and properties, and severely injure people.

During the design and safety assessment of coastal flood defenses, wave runup^9^ and/or wave overtopping^10,11^ are the main parameters to define its height. Wave overtopping is usually characterized by the average of instantaneous discharges per linear meter of width and can be expressed in m^3^/s per m or l/s per m^10^. The overtopping criteria for a design highly depend upon the function of the structure and the degree of protection required^11^. In fact, large wave overtopping can be acceptable in some locations such as isolated breakwaters or jetties where access can be restricted without major disturbance to the community. However, this is not the case with sea defense structures such as seawalls, promenades, or boulevards on urban beaches where the utilization of these structures (functional safety) demands more restrictive limits of wave overtopping than structural safety^12^. Ideally, managers of these structures should allow the occurrence of direct hazards from wave overtopping only when appropriate action plans to reduce risks are implemented ahead of extreme events^7^, but this is nota frequent practice. The definition of the hazard severity depends upon exceeding admissible levels of wave overtopping, which would trigger specific impacts on material assets and people. However, these admissible levels are not unanimous so far and large variability is found in the literature^7,10,12–14^.

Due to the lack of standarization and uniformity between previous works regarding the definition of critical situations and the associated wave overtopping limits, the present study aims at creating a conceptual impact level categorization that defines overtopping discharge thresholds and establishes warning levels for different types of exposed coastal elements. To reach this goal, this paper proposes a new and unified conceptual and quantitative categorization of warning levels for pedestrians and assets based on both coastal impact evidence collected during wave overtopping episodes with varying intensity and the capability of the existing assessment methods in predicting critical situations. The accuracy of the new proposed discharge limits in predicting warning levels was assessed for validation and consistency. The developed method was investigated and applied on two urban sandy beaches under intense development on the western and southern coasts of Portugal, Costa de Caparica (CC), and Praia de Faro (PdF), respectively, both protected by a hybrid system (beach face and sloping structure). At Costa da Caparica, two locations were investigated, CC1 (Santo António beach) and CC2 (Tarquineso-Paraiso beach). At these locations, only pedestrians and urban components and buildings are exposed at the crest of the structure (emergency vehicles are not considered here) (Table 1). Moreover, only at CC1, pedestrians and private vehicles have access to the area behind the promenade (Table 1). At Praia de Faro, the assessment involved pedestrians, vehicles and urban components and buildings (Table 1).

**Table 1:** Exposed coastal elements evaluated at each site.

Coastal element	Study sites		
	Costa da Caparica		Praia de Faro
	CC1	CC2	PdF
Pedestrians at the seawall crest/promenade	×	×	×
Urban components and buildings at the seawall crest/promenade	×	×	×
Vehicles at the seawall crest/promenade—low speed	–	–	×
Pedestrians behind the seawall with no clear view of the sea	×	–	–
Vehicles behind the seawall with no clear view of the sea—low speed	×	–	–

## Results

### Conceptual impact level classification

A new classification of wave overtopping impacts (and the associated discharge limits) on three coastal elements with high relevance in urban beach areas, namely pedestrians, urban components and buildings, and vehicles, hereafter called EW-Coast, was created. The unified warning level classification involves four warning levels based on the severity of the damage, from no impact (green color) to high impact (red color), with two intermediate levels (yellow and orange), as displayed in Table 2.

**Table 2:**
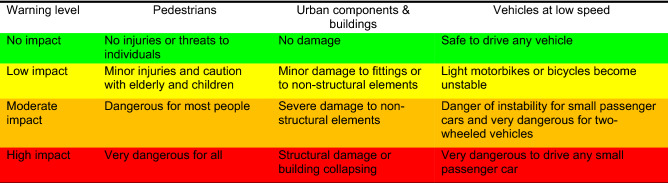
Unified warning level definition.

For pedestrians, no impact means that wave overtopping does not induce injuries or threats, even for the most vulnerable population, e.g., the elderly and children. Low impact implies that only the most vulnerable population can suffer minor injuries that do not threaten their lives. Moderate impact indicates that most people can be exposed to a fall due to friction instability (slipping) or moment (toppling) or being hit by debris, and only an alerted and active person can avoid the damage. High impact implies unsafe to everyone.

Regarding the urban components and buildings, no impact represents that the urban components, namely fittings, posts and non-structural elements, such as windows and doors, can resist the flow impacts without any damage. For low impact, those elements are marginally damaged, while for moderate impact, they are severely damaged. High impact implies structural damage or building collapse.

**Figure 1:**
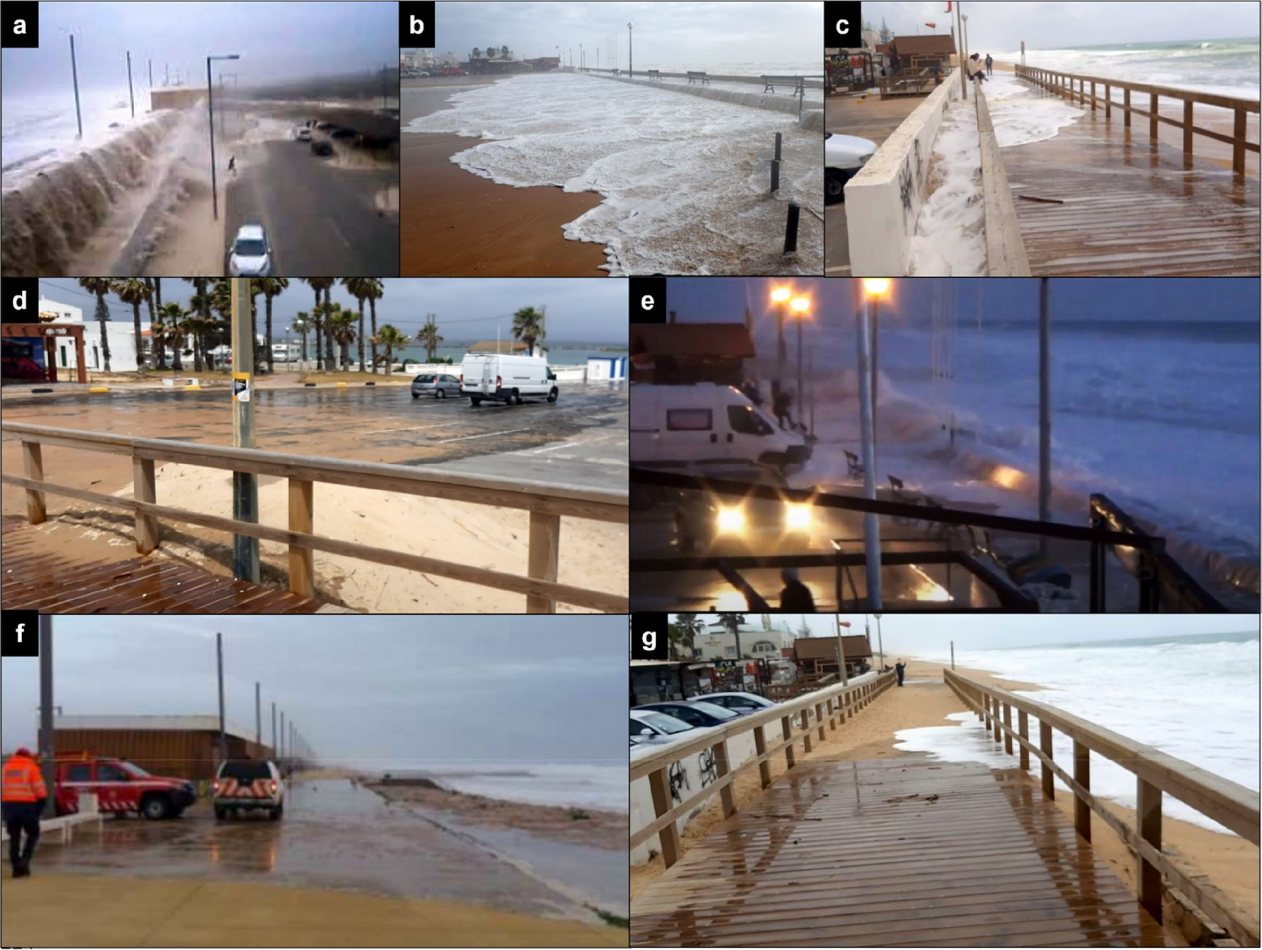
Images collected at the study sites that illustrate examples of different warning levels. (**a**) High impact for vehicles and pedestrians behind the promenade, and high impact for pedestrians and moderate impact for urban components and buildings at the crest at CC. The location corresponds to the beach cell CC1 (https://www.youtube.com/watch?v=WoLYcgO5F3g). (**b**) High impact for pedestrians and vehicles and moderate impact for urban components and buildings at PdF (credit: Agência Portuguesa do Ambiente, APA). (**c**) Low impact for urban components and buildings and vehicles and high impact for pedestrians at PdF (credit: authors). (**d**) Low impact for urban components and buildings and vehicles and high impact for pedestrians at PdF (credit: authors). (**e**) High impact for pedestrians and moderate impact for urban components and buildings and vehicles at PdF (https://www.youtube.com/watch?v=IZyEuhg8ofo). (**f**) No impact for urban components and buildings and vehicles and low impact for pedestrians at CC1 (credit: APA). (**g**) No impact for urban components and buildings and vehicles and moderate impact for pedestrians at PdF (credit: authors).

For vehicles at low speed, no impact means that any vehicle can be driven safely. Low impact denotes that the overtopping flow can destabilize some very light vehicles, such as bicycles and light motorbikes, and/or can scare unaware drivers, not expecting to get wet, interfering briefly with their capacity to drive. The moderate impact means that small passenger cars, whose mass is equal to or lower than 1000 kg^15^, might become unstable and/or drivers may temporarily lose control of their vehicle if water hits the front window. The high impact implies that any conventional passenger car (two-wheel drive) will experience slipping and it is very dangerous to drive. Several examples from the study sites (CC and PdF) illustrating critical situations within the considered warning levels (Table 2) are depicted in Fig. 1.

The categories of the critical levels proposed by the existing assessment methods were reclassified (see Table 3) to agree with the unified definition shown in Table 2. The methods included here were Coastal Engineering Manual^13^ (hereafter CEM), EurOtop 2018^7^ (hereafter EurOtop), HIDRALERTA^14,16^, and FLOODsite^17^. It is important to state that some works, e.g., EurOtop only defines two levels of impact, tolerable and not tolerable, and therefore this method can be only considered in two warning categories as shown in Table 3. For this case, the impact levels of EurOtop were categorized as green and orange for pedestrians, green and red for urban components and buildings, and green and orange for vehicles (Table 3). For CEM, the four critical levels for pedestrians were adapted to agree with the classification presented in Table 2. Also, the orange level was omitted for urban components and buildings because only three impact levels are described in this manual for these assets. Moreover, CEM establishes specific limits for vehicles that are driven at low speed (here at the crest or behind the seawall depending on the study site, Table 1). Only two levels, green and orange, were considered for this asset (Table 3). For HIDRALERTA, four categories of warnings were used for pedestrians, urban components and buildings, and vehicles. FLOODsite considers four impact levels for pedestrians, which can be fitted in the warning gradation proposed in Table 2, while for the remaining elements, the low impact level was not considered (Table 3).

**Table 3:**
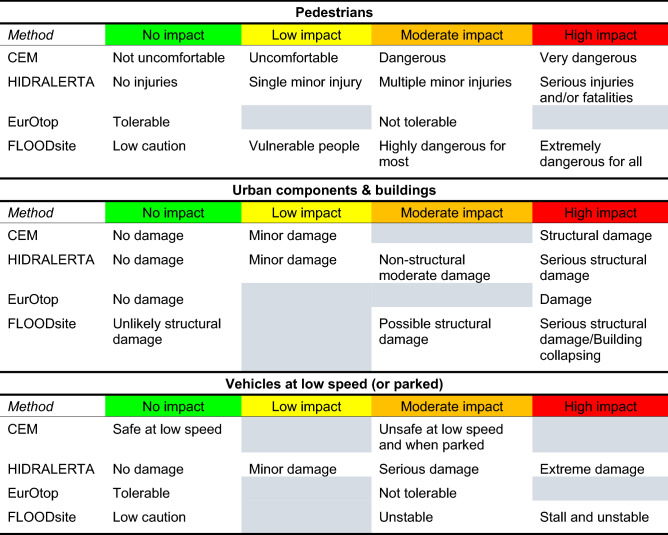
Warning levels of the four hazard assessment methods (CEM, HIDRALERTA, EurOtop and FLOODsite) adapted to the categorization displayed in Table 2. The grey cells indicate that the specific level is not defined or cannot be applied.

### Existing methods assessment

**Figure 2:**
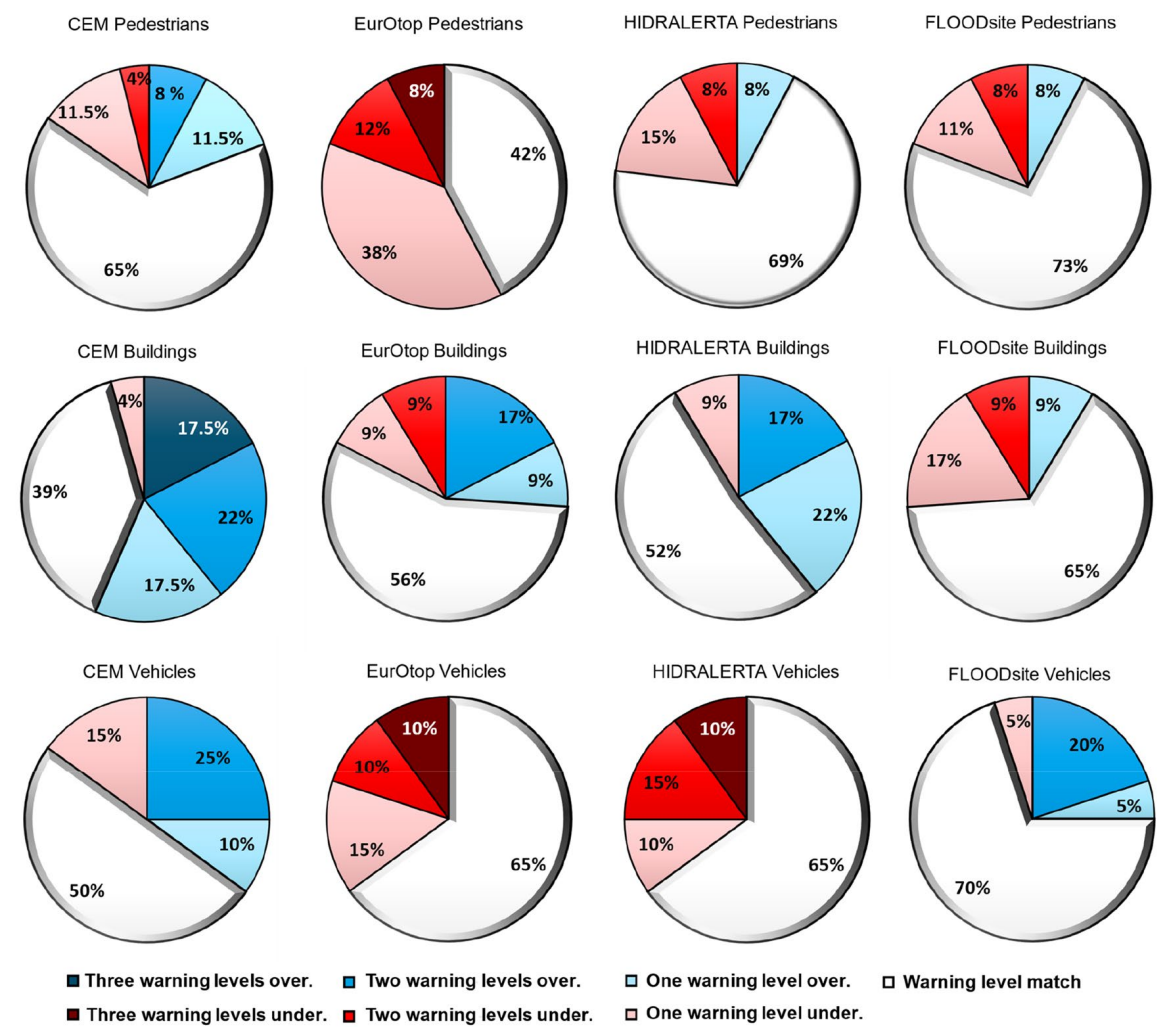
Comparison of the impacts predicted by the existing methods and observed for pedestrians, urban components and buildings, and vehicles at the evaluated sites. The results are presented in the form of the percentage of predicted cases that agree with the observations, overpredict (higher impact than observed) or underpredict (lower impact than observed). Warm colors represent underpredictions and cold colors overpredictions.

The mean wave overtopping discharge induced by several storm events (Supplementary Table S1 in the Supplementary material) was simulated by a process-based numerical model (Supplementary Table S2) and used to estimate the warning levels for pedestrians and coastal assets based on the limits shown in Supplementary Table S3 (CEM, HIDRALERTA and EurOtop) and Supplementary Table S4 (FLOODsite) in the Supplementary material. In total, 23 conditions from six storms distributed through two locations at CC and one location at PdF were simulated (Table 1, and Supplementary Tables S1 and S5). The obtained impact levels were then compared against observed impacts for analysis and validation. From the comparison between the predicted and observed impacts, it was found that: (1) Regarding the ability to predict impacts on pedestrians, FLOODsite displayed the best skills, as 73% of the overtopping episodes were well prognosticated against 42% for EurOtop, with the lowest skills (Fig. 2). Moreover, EurOtop’s predictions underestimated the observed impacts in 58% of the cases. HIDRALERTA and CEM exhibited similar ability to anticipate the impact levels (69% and 65% of the episodes were well predicted, respectively), with the former method underpredicting 15.5% of the cases against 23% by the latter method (Fig. 2). (2) Regarding urban components and buildings, FLOODsite was also the best of the existing methods, with 65% of the cases well predicted, against the worst estimates from CEM, which only agreed with the observations in 39% of the cases and overestimated the observed impacts in 57% of the episodes (39.5% overestimations in more than one warning level) (Fig. 2). EurOtop’s estimations agreed well with the observations in 56% of the cases and HIDRALERTA’s in 52%, with both methods overpredicting the observed impacts on 26% and 39% of the cases respectively (Fig. 2). (3) Regarding vehicles, FLOODsite predicted correctly 70% of the cases, EurOtop and HIDRALERTA 65%, and CEM 50%. When analyzing the general performance of the existing methods (the three exposed elements together), discrepancies between predicted and observed impacts in more than one level (under and overprediction) accounted for 12% for FLOODsite, 22% for EurOtop, 17% for HIDRALERTA, and 25% for CEM (Fig. 2).

### Unified impact level threshold definition

The assessment displayed in Fig. 2 highlighted that there was no single method capable of providing robust and reliable estimations for all considered levels and coastal elements. The new proposed unified categorization, besides being conceptual (Table 2), must also be translated into thresholds that distinguish different warning levels for each type of exposed element. The newly proposed association between wave overtopping discharges and each warning level (EW-Coast method) for the considered exposed elements is presented in Table 4. The critical wave overtopping discharge limits were defined based on the assessment of the existing methods and the impact observations. The limits vary depending on the resistance and vulnerability of the elements. For instance, for pedestrians, low and high impact warnings require discharges of at least 0.03 and 1 l/s per m respectively, while for urban components and buildings, the same warning levels require discharges of at least 1 and 20 l/s per m respectively.

**Table 4:**
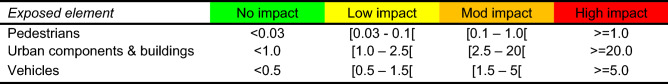
Unified mean discharge limits (l/s per m) of EW-Coast to split between different warning levels.

### Unified impact level threshold assessment

**Figure 3:**
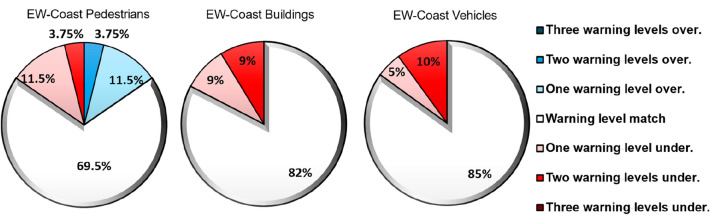
Comparison of the impacts predicted by the unified method (EW-Coast) and observed for pedestrians, urban components and buildings, and vehicles at the evaluated sites. The results are presented in the form of the percentage of predicted cases that agree with the observations, overpredict (higher impact than observed) or underpredict (lower impact than observed). Warm colors represent underpredictions and cold colors overpredictions.

The same mean wave overtopping discharge computations used in the previous assessment were considered to estimate the warning levels for the pedestrians and coastal assets based on the limits shown in Table 4. Based upon Fig. 3, the comparison between the predicted and observed impacts revealed that: (1) Regarding pedestrians, EW-Coast, correctly predicted 69.5% of the overtopping episodes, while the majority of the situations (92.5%) agreed with the observed impact or were within one level of difference (overestimation or underestimation) with respect to the observations. (2) Regarding urban components and buildings, EW-Coast identified 82% of the cases (Fig. 3), largely overcoming the performance of existing methods, as shown in Fig. 2. (3) Regarding vehicles, 85% of the cases were well-predicted by EW-Coast overcoming all the existing methods. For this asset, 5% of the cases underestimated the observations in one level and 10% in two levels. Moreover, for the three exposed elements together, only 9% of the predicted cases presented a difference with the observations of more than one warning level.

## Discussion

Coastal structures are rarely designed for zero overtopping during extreme storms^10^ and therefore managers must be prepared to implement specific risk-reduction measures when wave overtopping exceeds acceptable limits that jeopardize the functional safety of seawalls, promenades, or boulevards^10^. However, as demonstrated, the existing methods do not provide a standard description of potential impacts on pedestrians and coastal assets related to urban beaches and differ in the predicted warning level for the same event and exposed element. That is most probably related to the large variability among those methods regarding the discharge limits that induce critical situations and their severity. Therefore, varying predictive skill was found among the existing methods, with most of them presenting large percentages of underprediction or overprediction of the observed impacts (Fig. 2). It was found that overall, the existing methods failed in correctly predicting the warning level in between 27 and 61% of the cases. Moreover, the average percentage of failure considering together the three types of exposed elements was 48% for CEM, 45% for EurOtop, 38% for HIDRALERTA and 30% for FLOODsite. A systematic underprediction might not anticipate impacts and a frequent overprediction can release false alarms that will discredit the predictions.

Under the demand for a clear and unified flood level characterization with verified limits that can be applied to urban beaches, a classification of impact levels for pedestrians, urban components and buildings, and vehicles that integrates all existing methods is presented. The assessment of the new proposed limits revealed that they overcame the existing methods in predicting the warning levels with the erroneous predictions being reduced to an average (three exposed elements) of 21%, indicating an improved prediction between 27 and 9% with respect to the methods with the lowest and highest skills. Furthermore, when comparing against the existing method with the highest skills (FLOODsite), EW-Coast reduced the erroneous predictions by 17% and 15% in urban elements and buildings, and vehicles respectively while both methods obtained a similar percentage of failures for pedestrians (~ 30%).

The limits proposed here for pedestrians are within the same order of magnitude as those for HIDRALERTA. These limits are also in line with others used in early warning systems namely Triton in England^18^, which establishes the lowest level of warning when mean overtopping exceeds 0.1 l/s per m. This value was inspired by the perceived overtopping threshold documented by Belloti et al*.*^19^ on a field survey. For vehicles, the new limits are in line with those proposed in EurOtop (when the incident significant wave height, H_m0_, at the toe of the structure exceeds 3 m). Conversely, the limits proposed for urban components and buildings are higher than the existing methods and this promoted a better agreement with the observations, while the other methods highly overestimated the impacts. Therefore, the unified mean discharge limits proposed in this study, were inspired by a review of the pre-existent knowledge generated by well-known methodologies (Coastal Engineering^13^, EurOtop 2018^7^, HIDRALERTA^14^ and FLOODsite^17^) and altered based on field observations enabling the creation of a more robust classification that better characterizes the wave-induced impacts.

In the present study, for each condition, wave overtopping was simulated five times with the process-based model XBeach^20^ (non-hydrostatic mode) by using nominally identical JONSWAP wave spectra parameters, but randomly varying the initial seed value. Instead of the minimum or the average values, the maximum value of mean discharge among the five simulations was selected to characterize the hazard as it was observed that, in general, this value resulted in the best agreement with the observations for the assessment methods investigated. The overtopping estimations of physical experiments and numerical models simulating the propagation and decay of individual waves (e.g. XBeach^20^ and SWASH^21^) are subject to uncertainties due to the inherent randomness of the overtopping process. In fact, McCabe et al.^22^ declared that wave overtopping was very sensitive to changes in the initial seed and the overall overtopping rates can differ by up to a factor of two for nominally identical wave conditions. Williams et al*.*^23^ noticed that variability due to the seeding was larger for low overtopping conditions. Furthermore, the physical phenomenon of overtopping is affected by the specific sequence of waves that arrives at the shore^24^. However, as the phase distribution cannot be predicted, this adds uncertainty to any method of obtaining overtopping predictions^23,25^. There are other less complex methods to simulate wave overtopping, including empirical formulas^7,26^ that neglect that uncertainty, unless they are used within a probabilistic approach. However, they include some simplifications (for instance the beach profile) and the infragravity energy is not considered, while the effects of the stochastic uncertainty of the random phasing can be very relevant when predicting overtopping discharges^25^.

Collecting overtopping measurements in the field is very challenging^27^, and therefore, information to conduct a proper validation against quantitative data of any of the different methods to estimate overtopping in real conditions is very scarce^25^. Therefore, the comparison and validation of overtopping critical limits against observed data have rarely been investigated. Here, the high level of agreement with the impact observations and the ability of the predictive method to respond to varying storm conditions (from no-overtopping to remarkably high discharge values) guarantee a good accuracy that relates correctly the impact with the existing physical processes. Moreover, numerical models based on the non-linear shallow water equations with a pressure correction term, such as XBeach^20^ (non-hydrostatic mode) and SWASH^21^ are rapidly increasing their utilization for similar analyses confirming their suitability for wave overtopping studies^25^. Importantly, XBeach has shown good accuracy skills in simulating wave runup and mean overtopping discharges in dikes fronted by a shallow foreshore^28^ with similar characteristics to the cross-shore profiles assessed here. It is important to remark that the variability of the wave overtopping estimations provided by different methods (numerical models, empirical formulas and neuronal networks) can be very important^29,30^ as well as the subsequent impact predictions. However, this short of uncertainty depends on the methods used for the hazard computations and not on the proposed thresholds categorizing the warning levels.

In the present study, the feedback mechanisms between beach morphology and wave runup are not simulated and this can represent a major limitation. While feedback mechanisms are not expected in the upper part of the studied areas (man-made rigid structures), these can be relevant at the fronting beach profile. Previous studies^31–33^ have demonstrated that the morphological evolution of the sandy bottom highly affects the maximum wave runup, the time-integrated total wave overtopping volume and the timing of peak wave overtopping. While the effect of the morphological evolution can affect wave overtopping, most available methods to simulate wave-induced floodings, such as empirical formulas^26,34^, neuronal network tools^35^, and specific numerical models^20,21,36,37^, neglect the morphodynamic response. However, if there are strong morphodynamic feedbacks between beach morphology and runup incursions, then these methods are no longer valid and other alternatives might be required (e.g. XBeach^20^ surfbeat or IH2VOF-SED^38^). Another source of uncertainty is the lack of pre-storm beach topography measurements to be implemented in the numerical model for each storm. Instead, representative pre-storm conditions (from topographical surveys) were implemented in the numerical model. The existence of an unusually large berm width or beach scarp can affect the runup propagation, especially for low overtopping conditions^39^.

In areas with an energetic wave climate, watching violent waves breaking and overtopping can promote considerable public interest^40^ increasing the possibility of injuries to people and damage to vehicles, at the same time that buildings or urban elements can be affected. Therefore, authorities should adopt risk-reduction measures to minimize damage or even fatalities associated with overtopping hazards. If the new verified and unified impact characterization is implemented in operational and warning systems, decision-makers will be informed in advance about potential flooding-induced damages, being able to activate risk-reduction measures that safeguard pedestrians and assets on coastal defenses, promenades, or boulevards on urban beaches. It is important to remark that these thresholds are only applicable in areas with similar settings to those evaluated here (i.e., excluding high vertical structures where impulsive, violent, and sudden overtopping may occur, with falling or high-velocity jets). This integrated classification will also support the implementation of alert systems endorsing the fulfillment of the priorities defined in the Sendai Framework for Disaster Risk Reduction 2015–2030 and the United Nations Sustainable Development Goals^41^ (1, 3, 8, 11, 13 and 14).

## Methods

### Wave overtopping assessment studies

To reach the main aim of the paper—proposing an integrated impact level classification (conceptual and quantitative) for overtopping at urban beaches—a comprehensive and integrative analysis of the existing methods defining critical limits to overtopping impacts was performed. To the best knowledge of the authors, only Coastal Engineering Manual^13^, EurOtop 2018^7^, HIDRALERTA^14^ and FLOODsite^17^ report wave overtopping indicative thresholds to characterize hazards on the actors involved in the functional safety of the structure namely pedestrians, buildings (structural and non-structural components), and vehicles. The Coastal Engineering Manual^13^ presents a table with critical values of average overtopping discharges that condenses the information from previous field and full-scale test experiments developed in Europe^11,42,43^ and Japan^44–47^. EurOtop, in its last version released in 2018^7^ updated the limits of overtopping for pedestrians presented in EurOtop 2007^10^. In the older version, a limit of 0.1 l/s per m was established for pedestrians aware of the hazards with a clear view of the sea. This limit was derived mainly from observations^11,44,48,49^ and additional measurements of the CLASH project^40,50^. A further precautionary limit of 0.03 l/s per m was also suggested for unusual conditions where pedestrians have no clear view of incoming waves; may be easily upset or frightened as they are not dressed to get wet; may be on a narrow walkway or close proximity to a drip or fall hazard (not all of these conditions are required simultaneously). In the newer version, the authors defined three limits for overtopping for pedestrians at the crest of a structure with a clear view of the sea and considering that the expected overtopping is neither sudden nor violent based on estimations and measurements of overtopping at two breakwaters (Oostende and Ostia) and physical experiments^7^. Those limits are 0.3, 1, and 10–20 l/s per m for incoming H_m0_, at the toe of the structure of 3, 2 and 1 m, respectively, as it is considered that even for the same mean discharges, higher waves promote higher maximum volumes. In the present study, at CC the toe of the structure was considered at the intersection between the foreshore and the seawall, while at PdF as the structure is buried the toe of the structure was considered as the transition between the low tide terrace and the foreshore. For vehicles, in EurOtop 2018^7^, the tolerable limits no longer discriminate between low and high speeds (as was specified in EurOtop 2007^10^) and three tolerable limits are presented: 5, 10–20 and 75 l/s per m, depending on the H_m0_ value at the toe of the structure. It is assumed that these vehicles are driven in an area with access to people and thus, the speed is not high. The tolerable limit proposed in EurOtop 2018^7^ for structural components of buildings is 1 l/s per m and it is based on estimations of overtopping at the Belgium coast. Importantly, impact gradation is not considered for any of the elements introduced. HIDRALERTA^14,16^ establishes four impact categories, no impact, minor, serious and extreme, and the associated mean wave overtopping limits for different coastal elements. The limits that the authors provide to categorize the impacts are based on recommendations from EurOtop 2007^10^ and observations reported by coastal and port authorities in a limited number of coastal areas and harbors in Portugal. While HIDRALERTA was initially devoted to port activities, some actors, such as pedestrians, vehicles and buildings, can be also present on urban beaches and therefore, they were also considered on the current study. For pedestrians, HIDRALERTA separates between aware and unaware people. For aware pedestrians, the minor impact category is triggered when the mean overtopping reaches 0.1 l/s per m and the extreme category is activated when the discharge is at least 1 l/s per m. For unaware pedestrians, the limits are smaller, between 0.03 l/s per m for minor impact, and 0.3 l/s per m for extreme impact. The limits established for buildings are lower than 0.1 l/s per m (no damage), between 0.1 and 0.4 l/s per m (minor damage), between 0.4 and 1 l/s per m (non-structural moderate damage) and 1 l/s per m (serious structural damage). The values that define each impact level were selected based on the coastal and port managers’ experience. FLOODsite^17^ developed the model risk to life from flooding in Europe. This model underlines the consequences of flooding at different depths and velocities using the maximum depth-velocity product per linear m as an indicator (equivalent to maximum instantaneous discharges for overtopping episodes). It was constructed based on work already undertaken in the United Kingdom and new data collected on flood events in Continental Europe^17^. It is important to declare that this study only gathered damage data related to fluvial flooding, and coastal environments were not included. Therefore, the considered limits are an adaptation to overtopping induced damages. As suggested by the FLOODsite authors’, when comprehensive information about the exposed elements is not available, the medium vulnerability category was selected. The values of maximum depth-velocity product to characterize the considered impact levels for pedestrians, buildings and vehicles were adapted from their Fig. 2(6). The model offers four different levels of hazards: low, medium, high and extreme. However, for buildings and vehicles, only three levels were considered here. For pedestrians and vehicles, depth-velocity reaching 0.25 m^3^/s per m results in critical situations. When the depth-velocity is equal to or higher than 1.1 m^3^/s per m represents extreme damage for pedestrians and possible structural damage in buildings. All this information is summarized in Supplementary Tables S2 and S3, which were adapted from the original methods.

### Study cases

**Figure 4:**
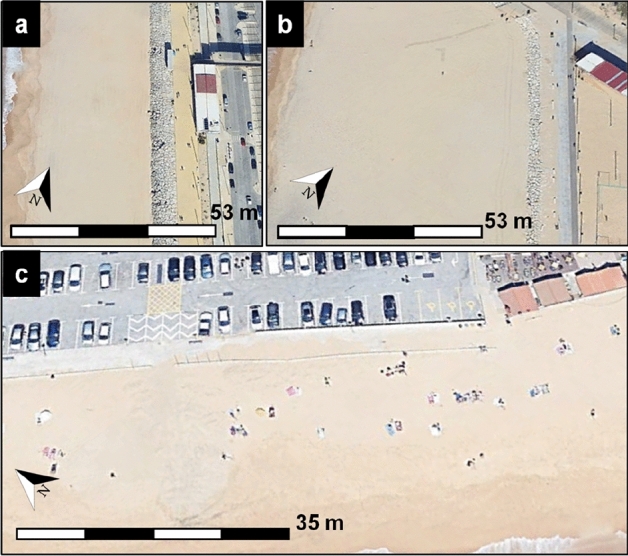
(**a**) Cell CC1 at Costa da Caparica. (**b**) Cell CC2 at Costa da Caparica. (**c**) Praia de Faro.

The new warning level description with the associated unified limits was developed and tested at two coastal areas in Portugal with contrasting wave regimes. Costa da Caparica (CC) is a mesotidal (dominated by semidiurnal constituents), sandy, urban beach located in the municipality of Almada, on the west coast of Portugal, a few kilometers south of the outer Tagus estuary^51^. This urban area is under intensive human pressure with several touristic and economic activities located on the seawall crest^51^. The coastline was severely modified with hard defense structures such as sloping rock armour seawalls (Fig. 4), which avoid its retreat, and a groin field. The groin field creates six sandy cells with an alongshore length that varies between 250 and 400 m. Two of these cells were evaluated here, CC1 (Santo António beach) and CC2 (Tarquineo-Paraiso beach) (Fig. 4). Cell CC1 is characterized by a 145 m beach width (from the seawall toe to the mean sea level, MSL, shoreline) at its central part (average conditions) and the seawall crest is 6.36 m above MSL. Cell CC2 has a wider beach, around 170 m (from the seawall toe to the MSL shoreline) for average conditions, and the elevation of the seawall crest is 6.04 m above MSL. In contrast to CC1, at this beach cell, the seawall crest has the same elevation as the surrounding landscape and thus, there is no shift in elevation in the area behind the crest (Fig. 4).

Praia de Faro (PdF) is located on the Ancão Peninsula, the westernmost sector of the Ria Formosa barrier island system, on the southern coast of Portugal^52^. This narrow sand peninsula separates the Atlantic Ocean and a coastal lagoon and it is subject to a semidiurnal and mesotidal regime^52^. The section considered in this study is under intense urban development where the natural defense, namely the dune, was completely removed (Fig. 4) and the oceanfront is stabilized with a buried rip-rap seawall^52^, and a partially buried short wall (less than half a meter). These structures protect a 3 m-wide walking wooden path that gives access to the urbanized area with a large parking lot and several restaurants and cafes (Fig. 4). The beachfront promenade, accessible to pedestrians is 5.2 m above MSL, and the beach width is approximately 40 m, from the wall limiting the bathing area to the MSL shoreline.

### Storms

In order to assess the accuracy of the methods in predicting storm impacts for pedestrians and assets, several storms with different levels of intensity were selected as shown in Supplementary Table S1. In-situ observations and videos from YouTube were collected (see some examples in Fig. 1) during these storms and then, based on the authors’ judgment, the induced impact levels were classified according to Table 2. On the two beach cells from CC, storm Hercules 2014, which brought severe damage along the entire Portuguese coast, storm Emma 2018, and another storm in 2017, hereafter referred to as Feb2017, were used to compare the predictions of the methods and the observations. In PdF, five storms were selected: storm Hercules 2014, storm Emma 2018, storm Elsa 2019, storm Barbara 2020, and storm Lola 2021, where for the first and the last storms more than one sea-state were included in the analysis (Supplementary Tables S1 and S4). The oceanic and atmospheric conditions during these storms used as input information for the wave overtopping computations were extracted from official systems (ERA-5 hindcast and Puertos del Estado), XTIDE (www.flaterco.com/xtide) and nearby observations (tidal gauge and wave buoy) (see modeling description below). The wave and sea level conditions during these storms in front of both sites are shown in Supplementary Table S1.

### Wave overtopping modeling

To model wave overtopping at CC, a numerical framework was used consisting of SWAN^53^ + XBeach^20^. The SWAN model used a nested approach with two structure grids to simulate wave processes from deep water to the offshore boundary of the XBeach model (~ 22 m depth). The outer grid wave model had a resolution of 500 m (both in the cross-shore and along-shore directions) and it was forced along the domain boundaries with wave data from ERA-5^54^. Wind-transferred energy processes were also considered in the wave modeling by including temporally and spatially variable wind speed and direction information provided by ECMWF. The inner grid wave model was forced along the domain boundaries with wave information provided by the outer grid and it had a resolution of 100 m in both directions. The sea-bottom elevation data was extracted from two sources^55,56^. The default values of the model parameters were used. Then, two one-dimensional non-hydrostatic XBeach models (CC1 and CC2) were used to simulate nearshore processes of individual waves, short and long wave motions, run-up incursions, vertical exchange of ground and surface water, wave overtopping and flood propagation. However, these two models did not include geomorphological processes. The flow boundary conditions were set to nonh_1d in the front and abs_1d in the back and Neumann conditions were selected for the lateral boundaries. The CFL was set to 0.55. A two-layer scheme in the vertical with the layer distribution set to the default value (0.33) was applied to better simulate the dispersion relation and shoaling of waves in intermediate depths^57^. The parameter maxbrsteep that governs the maximum wave steepness criterium was set to 0.4. A constant manning coefficient of 0.02 was chosen to represent the bottom roughness. The grid resolution varied between 4 m (offshore) and less than 0.5 m in the nearshore and emerging areas. The nearshore and beach face elevations were extracted from surveys performed by the COSMO program (https://cosmo.apambiente.pt/) and they were considered to be representative of average conditions. The elevation of the seawall and the urbanized area were extracted from a survey conducted by the authors. These simulations used as input oceanic conditions the water level measurements from the tidal gauge at Cascais and the wave information obtained from the inner grid wave model. After a warm-up period, XBeach simulated 600 waves that multiplied by their mean period resulted in the time used to average the instantaneous wave overtopping discharges. Discharge values were extracted at the interest locations (the seaward and landward edge of the seawall crest and behind the promenade, as displayed in Table 1 and Supplementary Table S2) every half second, for both CC1 and CC2. To account for the stochastic effects of the wave overtopping process, the simulations were repeated five times with identical static water levels and wave parameters, but randomly varying the seed number used to generate the free surface elevation and velocity time series at the seaward boundary. The mean discharge used to assess the impact levels corresponded to the maximum among the five simulations.

The modeling approach used in PdF involved the same numerical models. The SWAN model consisted of one structured grid that covered the entire southern Portuguese coast with an approximated resolution of 350 m and 600 m in the cross-shore and alongshore directions, respectively. The sea-bottom elevation was extracted from the open dataset MIRONE^58^. This model was forced with wave information provided by buoy measurements or numerical modeling from Puertos del Estado (https://www.puertos.es/en-us/oceanografia/Pages/portus.aspx) imposed along the offshore model boundary that laid approximately at the 100 m bathymetric contour line. Using model default values, the wave conditions were propagated until 20 m depth, approximately, where the XBeach offshore boundary was located. Besides the wave conditions propagated to the model boundary, surge and tide levels from Huelva tidal gage and XTIDE respectively were used as input conditions for XBeach. Model parameters used in this model for PdF were similar to the parametrization used in CC and only maxbrsteep had a different value (0.5 instead of 0.4) to account for the particularities of this site. The grid resolution varied between 2 m (offshore) and 0.5 m (nearshore and emerging areas) and the sea-bottom and inland elevation was interpolated from several sources: a UAV survey conducted by the COSMO program that covered the emerged beach and the structure; a bathymetry survey performed by the COSMO program that measured the region from MSL until − 13.5 m MSL; and a regional bathymetry extracted from MIRONE^58^ that was used to obtain information below − 13.5 m MSL.

## Supplementary Information

Supplementary Information.

## Data Availability

Data used in this study are available upon request to the corresponding author*.*
