# Supplementary material for: Conceptual and quantitative categorization of wave-induced flooding impacts for pedestrians and assets in urban beaches
Source: Sci Rep. 2023 May 4;13:7251. doi: 10.1038/s41598-023-32175-6 (PMC10160089; doi:10.1038/s41598-023-32175-6)
Supplement: Supplementary file 1 — Supplementary Information. [file 41598_2023_32175_MOESM1_ESM.pdf]

Conceptual and quantitative categorization of wave-induced flooding impacts for pedestrians and assets in urban beaches

J. L. Garzon*(1), Ó. Ferreira(1), M.T.Reis(2), A. Ferreira(2), C.J.E.M. Fortes(2) and A.C. Zózimo(2)

(1) CIMA – Centre for Marine and Environmental Research, FCT, Universidade do Algarve, Faro, Portugal
(2) Hydraulics and Environment Department, National Laboratory for Civil Engineering, Av. do Brasil, 101, 1700-066 Lisboa, Portugal.

Supplementary material

Table S1. Oceanic conditions used on the XBeach simulations.

**Costa de Caparica**				
Storm	Hs (m)	Tp (s)	Direction (°N)	Water level (m)
Hercules	4.53	20.0	270	2.05
Emma	4.15	10.9	270	2.22
Feb2017	4.69	16.5	270	1.21
**Praia de Faro**				
Storm	Hs (m)	Tp (s)	Direction (°N)	Water level (m)
Hercules, 6th	1.37	20.5	240	0.95
Hercules, 7th	1.8	18.6	240	1.08
Emma	5.57	11.9	220	1.75
Elsa	4.35	9.8	230	1.1
Barbara	3.14	8.3	216	1.46
Lola, 24th, 11:00	2.95	14.7	225	0.51
Lola, 24th, 12:00	2.96	14.7	225	0.94
Lola, 24th, 13:00	2.97	14.7	224	1.19
Lola, 24th, 14:00	2.97	14.7	223	1.2
Lola, 24th, 15:00	2.95	14.7	223	0.97
Lola, 24th, 16:00	2.91	14.7	224	0.56
Lola, 25th, 02:00	2.30	14.7	229	1.46
Lola, 25th, 12:00	1.97	13.3	228	0.76
Lola, 25th, 13:00	1.93	13.3	228	1.23
Lola, 25th, 14:00	1.88	13.3	228	1.47
Lola, 25th, 15:00	1.83	13.3	228	1.39
Lola, 25th, 16:00	1.79	13.3	228	1.04

Table S2. Minimum, maximum and mean overtopping discharges and depth velocities at CC and PdF.

Costa de Caparica

**Storm**	**Profile**	Assessment location	**q (l/s per m)**			**Depth velocity (m^3/s per m)**		
			**[Mix – Max]**		**Mean**	**[Mix – Max]**		**Mean**
Hercules	CC1	Crest	3.510 -	5.721	4.549	3.870 -	7.222	4.922
		Buildings at crest	1.893 -	3.820	2.499	1.412 -	3.536	2.057
		Behind seawall	2.734 -	4.955	3.527	1.229 -	4.479	2.308
Hercules	CC2	Crest	0.803 -	3.028	1.460	1.753 -	3.803	2.817
		Buildings at crest	0.464 -	2.114	0.857	0.641 -	2.159	1.356
Emma	CC1	Crest	0.000 -	0.000	0.000	0.000 -	0.000	0.000
		Buildings at crest	0.000 -	0.000	0.000	0.000 -	0.000	0.000
		Behind seawall	0.000 -	0.000	0.000	0.000 -	0.000	0.000
Emma	CC2	Crest	0.000 -	0.000	0.000	0.000 -	0.000	0.000
		Buildings at crest	0.000 -	0.000	0.000	0.000 -	0.000	0.000
Feb2017	CC1	Crest	0.000 -	0.037	0.010	0.000 -	0.402	0.114
		Buildings at crest	0.000 -	0.000	0.000	0.000 -	0.000	0.000
		Behind seawall	0.000 -	0.000	0.000	0.000 -	0.000	0.000
Feb2017	CC2	Crest	0.000 -	0.007	0.001	0.000 -	0.083	0.017
		Buildings at crest	0.000 -	0.000	0.000	0.000 -	0.000	0.000

Praia de Faro

**Storm**	Assessment location	**q (l/s per m)**			**Depth velocity (m^3/s per m)**		
		**[Mix – Max]**		**Mean**	**[Mix – Max]**		**Mean**
Hercules, 6th	Promenade	0.000 -	0.200	0.054	0.010 -	0.490	0.136
	Park/Buildings	0.000 -	0.170	0.036	0.000 -	0.280	0.062
Hercules, 7th	Promenade	0.010 -	0.710	0.324	0.040 -	0.700	0.466
	Park/Buildings	0.000 -	0.600	0.272	0.000 -	0.410	0.260
Emma	Promenade	12.632 -	16.431	14.765	2.698 -	4.132	3.596
	Park/Buildings	11.449 -	15.140	13.610	2.637 -	4.321	3.554
Elsa	Promenade	0.078 -	0.405	0.199	0.207 -	0.721	0.431
	Park/Buildings	0.051 -	0.349	0.160	0.081 -	0.400	0.229
Barbara	Promenade	0.000 -	0.000	0.000	0.000 -	0.000	0.000
	Park/Buildings	0.000 -	0.000	0.000	0.000 -	0.000	0.000
Lola, 24th, 11:00	Promenade	0.000 -	0.000	0.000	0.000 -	0.000	0.000
	Park/Buildings	0.000 -	0.000	0.000	0.000 -	0.000	0.000
Lola, 24th, 12:00	Promenade	0.060 -	0.201	0.141	0.156 -	0.586	0.342
	Park/Buildings	0.009 -	0.157	0.101	0.022 -	0.352	0.164
Lola, 24th, 13:00	Promenade	0.069 -	1.524	0.770	0.176 -	1.633	0.964
	Park/Buildings	0.539 -	1.333	0.844	0.055 -	1.044	0.585
Lola, 24th, 14:00	Promenade	0.244 -	1.209	0.625	0.341 -	1.149	0.754
	Park/Buildings	0.136 -	1.076	0.486	0.146 -	0.805	0.409
Lola, 24th, 15:00	Promenade	0.000 -	0.225	0.066	0.000 -	0.352	0.122
	Park/Buildings	0.000 -	0.173	0.045	0.000 -	0.195	0.053
Lola, 24th, 16:00	Promenade	0.000 -	0.000	0.000	0.000 -	0.000	0.000
	Park/Buildings	0.000 -	0.000	0.000	0.000 -	0.000	0.000
Lola, 25th, 02:00	Promenade	1.031 -	1.934	1.553	0.649 -	2.339	1.163
	Park/Buildings	0.842 -	1.665	1.323	0.374 -	1.850	0.798
Lola, 25th, 12:00	Promenade	0.000 -	0.000	0.000	0.000 -	0.000	0.000

	Park/Buildings	0.000	-	0.000	0.000	0.000	-	0.000	0.000
Lola, 25th, 13:00	Promenade	0.000	-	0.067	0.013	0.000	-	0.116	0.023
	Park/Buildings	0.000	-	0.033	0.007	0.000	-	0.028	0.006
Lola, 25th, 14:00	Promenade	0.038	-	0.365	0.153	0.111	-	0.435	0.285
	Park/Buildings	0.016	-	0.275	0.104	0.028	-	0.229	0.135
Lola, 25th, 15:00	Promenade	0.000	-	0.002	0.000	0.000	-	0.007	0.001
	Park/Buildings	0.000	-	0.000	0.000	0.000	-	0.000	0.000
Lola, 25th, 16:00	Promenade	0.000	-	0.000	0.000	0.000	-	0.000	0.000
	Park/Buildings	0.000	-	0.000	0.000	0.000	-	0.000	0.000

Table S3. Adapted from CEM, HIDRALERTA and EurOtop 2018. Mean discharge limits in l/s per m.

**Aware pedestrians at the seawall crest/promenade**								
CEM				HIDRALERTA (based on EurOtop 2007)				EurOtop 2018
Not uncomfortable	*Uncomfortable*	*Dangerous*	*Very dangerous*	*No injuries*	*Single minor injury*	*Multiple minor injuries*	*Serious injuries or fatalities*	*Not tolerable*
<0.004	[0.004 - 0.03[	[0.03 - 1[	≥ 1	<0.1	[0.1 - 0.3[	[0.3 - 1.0[	≥ 1	> 0.3 / 1 / 10*
**Urban components & buildings**								
CEM				HIDRALERTA (based on EurOtop 2007)				EurOtop 2018
No damage	Minor damage	Structural damage		*No damage*	*Minor damage*	*Non-structural moderate damage*	*Serious structural damage*	*Non-tolerable*
<0.001	[0.001 - 0.03[	≥ 0.03		<0.1	[0.1 - 0.4[	[0.4 - 1.0[	≥ 1	≥ 1
**Unaware pedestrians behind the seawall crest/promenade**								
CEM				HIDRALERTA				EurOtop 2018
Not uncomfortable	*Uncomfortable*	*Dangerous*	*Very dangerous*	*No injuries*	*Single minor injury*	*Multiple minor injuries*	*Serious injuries or fatalities*	*Not tolerable*
<0.004	[0.004 - 0.03[	[0.03 – 1[	≥1	<0.03	[0.03 - 0.09[	[0.09 - 0.3[	≥0.3	> 0.3 / 1 / 10*
**Vehicles (low speed)**								
CEM				HIDRALERTA (low speed)				EurOtop 2018
Safe at all speeds		Unsafe at any speed and when parked		*No damage*	*Minor damage*	Serius damage	Extreme damage	*Non-tolerable*
<0.001		≥0.02		< 10	[10 -25 [	[25 - 50 [	≥50	≥ 5 / 10 / 75*

* Values corresponding to sea states with $Hm_0 \geq 3$ m / 2 m $\leq Hm_0 < 3$ m / 1 m $\leq Hm_0 < 2$ m

Table S4. Depth velocity in m^3/s per m adapted from FLOODsite

**Pedestrians**			
Low caution	Vulnerable people (e.g. children and the elderly)	Highly dangerous for most	Extremely dangerous for all
<0.25	[0.25 - 0.50[	[0.5 - 1.1[	>=1.1

**Vehicles**		
Low caution	Unstable	Stall and unstable
<0.25	[0.25 - 0.50[	>=0.5
**Buildings**		
Unlikely structural damage	Possible structural damage	Serious structural damage/Building collapsing
<1.1	[1.1 - 7[	>=7

Table S5. Observed and predicted impact levels for the coastal receptors at Costa da Caparica and Praia de Faro.

Costa de Caparica

Storm	Profile	Exposed elements	*Computed impact level*					*Observed impact level*
			CEM	EurOtop	HIDRALERTA	FLOODsite	EW-Coast	
Hercules	CC1	Pedestrians at the promenade	Red	Orange	Red	Red	Red	Red
		Urban components/buildings	Red	Red	Red	Orange	Orange	Orange
		Pedestrians behind the seawall	Red	Orange	Red	Red	Red	Red
		Vehicles behind the seawall	Orange	Green	Green	Red	Orange	Red
Hercules	CC2	Pedestrians at promenade	Red	Orange	Red	Red	Red	Red
		Urban components/buildings	Red	Red	Red	Orange	Yellow	Yellow
Emma	CC1	Pedestrians at the promenade	Green	Green	Green	Green	Green	Yellow
		Urban components/buildings	Green	Green	Green	Green	Green	Green
		Pedestrians behind the seawall	Green	Green	Green	Green	Green	Green
		Vehicles behind the seawall	Green	Green	Green	Green	Green	Green
Emma	CC2	Pedestrians at promenade	Green	Green	Green	Green	Green	Green
		Urban components/buildings	Green	Green	Green	Green	Green	Green
Feb2017	CC1	Pedestrians at the promenade	Orange	Green	Green	Yellow	Yellow	Yellow
		Urban components/buildings	Green	Green	Green	Green	Green	Green
		Pedestrians behind the seawall	Green	Green	Green	Green	Green	Green
		Vehicles behind the seawall	Green	Green	Green	Green	Green	Green
Feb2017	CC2	Pedestrians at promenade	Yellow	Green	Green	Green	Green	Green
		Urban components/buildings	Green	Green	Green	Green	Green	Green

Praia de Faro

Storm	Exposed elements	*Computed impact level*					*Observed impact level*
		CEM	EurOtop	HIDRALERTA	FLOODs	EW-Coast	
Hercules, 6th	Pedestrians at promenade	Orange	Green	Yellow	Yellow	Orange	Red
	Urban components/buildings	Red	Green	Yellow	Green	Green	Orange
	Vehicles in the parking lot	Orange	Green	Green	Orange	Green	Orange
Hercules, 7th	Pedestrians at promenade	Orange	Green	Orange	Orange	Orange	Red
	Urban components/buildings	Red	Green	Orange	Green	Green	Orange
	Vehicles in the parking lot	Orange	Green	Green	Orange	Yellow	Red
Emma	Pedestrians at promenade	Red	Orange	Red	Red	Red	Red
	Urban components/buildings	Red	Red	Red	Orange	Orange	Orange
	Vehicles in the parking lot	Orange	Orange	Yellow	Red	Red	Red
Elsa	Pedestrians at promenade	Orange	Green	Orange	Orange	Orange	Yellow
	Urban components/buildings	Red	Green	Yellow	Green	Green	Green
	Vehicles in the parking lot	Orange	Green	Green	Orange	Green	Green
Barbara	Pedestrians at promenade	Green	Green	Green	Green	Green	Green
	Urban components/buildings	Green	Green	Green	Green	Green	Green
	Vehicles in the parking lot	Green	Green	Green	Green	Green	Green

Lola, 24th, 11:00	Pedestrians at promenade						
	Urban components/buildings						
	Vehicles in the parking lot						
Lola, 24th, 12:00	Pedestrians at promenade						
	Urban components/buildings						
	Vehicles in the parking lot						
Lola, 24th, 13:00	Pedestrians at promenade						
	Urban components/buildings						
	Vehicles in the parking lot						
Lola, 24th, 14:00	Pedestrians at promenade						
	Urban components/buildings						
	Vehicles in the parking lot						
Lola, 24th, 15:00	Pedestrians at promenade						
	Urban components/buildings						
	Vehicles in the parking lot						
Lola, 24th, 16:00	Pedestrians at promenade						
	Urban components/buildings						
	Vehicles in the parking lot						
Lola, 25th, 02:00	Pedestrians at promenade						
	Urban components/buildings						
	Vehicles in the parking lot						
Lola, 25th, 12:00	Pedestrians at promenade						
	Urban components/buildings						
	Vehicles in the parking lot						
Lola, 25th, 13:00	Pedestrians at promenade						
	Urban components/buildings						
	Vehicles in the parking lot						
Lola, 25th, 14:00	Pedestrians at promenade						
	Urban components/buildings						
	Vehicles in the parking lot						
Lola, 25th, 15:00	Pedestrians at promenade						
	Urban components/buildings						
	Vehicles in the parking lot						
Lola, 25th, 16:00	Pedestrians at promenade						
	Urban components/buildings						
	Vehicles in the parking lot						
